# An Adaptive Intrusion Detection System in the Internet of Medical Things Using Fuzzy-Based Learning

**DOI:** 10.3390/s23229247

**Published:** 2023-11-17

**Authors:** Mousa Alalhareth, Sung-Chul Hong

**Affiliations:** 1 Department of Information Systems, College of Computer Science and Information System, Najran University, Najran 61441, Saudi Arabia; 2 Department of Computer and Information Sciences, Towson University, Towson, MD 21204, USA

**Keywords:** IoMT, IDS, LSTM, fuzzy logic, healthcare, deep learning

## Abstract

The Internet of Medical Things (IoMT) is a growing trend within the rapidly expanding Internet of Things, enhancing healthcare operations and remote patient monitoring. However, these devices are vulnerable to cyber-attacks, posing risks to healthcare operations and patient safety. To detect and counteract attacks on the IoMT, methods such as intrusion detection systems, log monitoring, and threat intelligence are utilized. However, as attackers refine their methods, there is an increasing shift toward using machine learning and deep learning for more accurate and predictive attack detection. In this paper, we propose a fuzzy-based self-tuning Long Short-Term Memory (LSTM) intrusion detection system (IDS) for the IoMT. Our approach dynamically adjusts the number of epochs and utilizes early stopping to prevent overfitting and underfitting. We conducted extensive experiments to evaluate the performance of our proposed model, comparing it with existing IDS models for the IoMT. The results show that our model achieves high accuracy, low false positive rates, and high detection rates, indicating its effectiveness in identifying intrusions. We also discuss the challenges of using static epochs and batch sizes in deep learning models and highlight the importance of dynamic adjustment. The findings of this study contribute to the development of more efficient and accurate IDS models for IoMT scenarios.

## 1. Introduction

The number of smart devices connected to the Internet, or the Internet of Things (IoT), is growing rapidly [1,2]. A substantial fraction of these devices are in the medical field, a trend known as the Internet of Medical Things (IoMT) [3,4]. The use of the IoMT has improved healthcare operations, remote services, and patient monitoring [5]. However, there are serious security and privacy issues as the IoT-enabled medical devices are vulnerable to a wide range of cyber-attacks [6,7,8,9]. If these devices are accidentally exposed, they could be exploited by adversaries using advanced persistent threats (APTs) and known weaknesses, potentially disrupting healthcare operations and endangering human lives [7,10]. Therefore, security should be a top priority when using the IoMT for remote health monitoring [2,9].

Detecting and mitigating attacks in the IoMT can be accomplished with various techniques and methods [1,2,11]. These include log monitoring, vulnerability management, threat intelligence, end device monitoring, and intrusion detection and prevention systems [12,13,14]. Intrusion detection systems, which rely on traffic anomalies, signature-based rules, or security policies, are frequently used to identify attacks in IoT-enabled networks [13,15]. However, traditional detection techniques often fall short as attackers continually refine their strategies and employ advanced hacking techniques [16,17,18]. For example, security policies can be circumvented if an attacker conducts network reconnaissance or reverse engineers network devices like routers and firewalls [19]. To enhance attack detection, researchers are exploring machine learning (ML) and deep learning (DL) solutions [16,20]. Thanks to advances in computing and processing capabilities, ML and DL techniques can be used on a large scale to predict attack events with greater accuracy.

While intelligent intrusion detection systems (IDSs) that use ML and DL techniques have been proposed [21,22,23], they may not be suitable for IoMT scenarios. These systems, designed for conventional networks, are not ideal for assessing attack detection in the IoMT because health IoT sensors connected to the Internet generate different types of data [24]. Additionally, in smart health applications, most existing methods only analyze network traffic to identify IoMT attacks, ignoring patient biometric information [24]. However, such information is crucial in the IoMT context as it offers insights into a patient’s condition and can be linked to network disruptions caused by attacks that affect the confidentiality, availability, and integrity of healthcare data [25,26]. Therefore, for more effective attack prediction, both network traffic and patient biometrics data should be considered together. Analyzing the relationship between these two disparate data types during an attack can provide a more comprehensive understanding of the situation. 

The existing literature presents a variety of deep learning-based intrusion detection systems (IDSs) for the Internet of Medical Things (IoMT) [25,27,28,29]. A common approach involves passing information of network flows and patient biometrics through several hidden layers of deep learning [10,16]. This approach employs a global attention layer for optimal feature extraction from the spatial and temporal characteristics of deep learning, and incorporates a cost-sensitive learning approach to address data imbalance [24]. However, these studies do not discuss the challenges related to the static number of epochs and batch sizes often used in deep learning models. Another study introduces a swarm-neural network-based model to detect intruders in IoMT systems [30]. This model acknowledges the security and privacy concerns that arise from transferring patient data to the cloud for processing, due to the limited storage and computation capacity of IoMT devices. However, the swarm-neural network model’s performance metrics are not clearly specified, and the concern of statically setting the number of epochs and batch sizes remains unaddressed. 

In the realm of explainable AI (XAI), a novel model, XSRU-IoMT [25], was proposed to detect sophisticated attack vectors in IoMT networks. This model leverages bidirectional simple recurrent units (SRUs) with skip connections to overcome the vanishing gradient problem and expedite the training process. While it improves the trust level by providing explanations for prediction decisions, the study does not offer insights on how the static number of epochs and batch sizes might influence the model’s efficiency and accuracy.

Another research proposes a cyber-attack detection method employing ensemble learning and a fog–cloud architecture [31]. This system uses a set of LSTM networks for initial learning and a decision tree for classifying attacks and normal events. While this paper offers an innovative framework for deploying IoMT-based approaches as cloud and fog services, it does not delve into the implications of setting a fixed number of epochs and batch sizes in the learning process. While various deep learning models have been proposed for IoMT intrusion detection, few discuss the impact of static epochs and batch sizes in training these models. Future studies might aim to dynamically adjust these parameters based on the data characteristics and model performance to potentially enhance the efficacy of the IDS in the IoMT.

The optimization of epoch numbers in deep learning models is contingent upon unique data characteristics, model architecture, and the specific tasks required. A popular technique for preventing model overfitting and enhancing the accuracy of new data is the use of ‘early stopping’. This process ceases training when there is a noted decline in model performance. Several research studies have applied early stopping methodologies to improve the precision of DL models. However, implementing early stopping methods in deep learning models poses challenges. A significant issue is the automated determination of the optimal stopping point, which can vary greatly depending on the data, model architecture, and task at hand. Striking a balance between mitigating overfitting and ensuring the model’s ability to generalize to new data is crucial. Moreover, the definition of performance degradation can vary depending on the dataset and task, complicating the application of early stopping methods across different contexts. Hence, there is an ongoing need for a more comprehensive understanding and application of early stopping in deep learning models. To this end, this paper is devoted to investigating the application of fuzzy logic for estimating the optimal value of the patience parameter used to trigger early stopping during the training phase of deep learning models. 

The proposed fuzzy-based self-tuning approach for intrusion detection in the Internet of Medical Things (IoMT) significantly advances the state of the art by introducing a dynamic early stopping mechanism tailored to the unique characteristics of IoMT data streams. This mechanism, underpinned by fuzzy logic, adaptively determines the optimal stopping point during training, a feature not commonly present in existing models, which often rely on static parameters. Furthermore, our self-tuning LSTM algorithm is specifically designed to address the challenges inherent in IoMT data, such as high dimensionality and the need for real-time processing, by autonomously adjusting the number of training epochs. This self-tuning capability is a substantial improvement from traditional methods that require manual epoch tuning. Additionally, our model’s integration of both network traffic and patient biometric data for intrusion detection is particularly innovative, as it leverages the correlation between these data types to provide a more nuanced detection capability in the IoMT context. An extensive experimental evaluation underscores the effectiveness of our approach, showcasing its competitive performance and improved adaptability in real-time threat detection scenarios. We believe these elements collectively underscore the novelty and improved efficacy of our proposed solution in the realm of IoMT security.

In particular, this study focuses on incorporating a dynamic early stopping approach into the Long Short-Term Memory (LSTM) classifier for the IDS in the IoMT. Recognizing this critical challenge, our paper is driven by the following specific objectives:

1. **To develop an intrusion detection system (IDS) tailored for the IoMT ecosystem:** We aim to design a system that not only detects common cyber-threats but is also capable of identifying IoMT-specific attacks that could disrupt healthcare services and compromise patient data.
2. **To implement a fuzzy-based self-tuning mechanism within an LSTM network:** Our objective is to enhance the traditional LSTM approach by incorporating a fuzzy logic component that dynamically adjusts the number of training epochs, thereby optimizing the model’s performance and responsiveness to the evolving IoMT threat landscape.
3. **To evaluate the effectiveness of early stopping techniques in deep learning models for the IoMT:** We seek to investigate how fuzzy logic can refine early stopping methods to prevent overfitting, ensure timely model convergence, and maintain a high detection accuracy.
4. **To assess the impact of integrating patient biometric data with network traffic analysis for intrusion detection:** Our research questions whether the inclusion of diverse data types can improve the IDS’s ability to detect sophisticated attacks within the IoMT framework.

By setting these objectives, we provide a clear roadmap for our research, guiding readers through the development and validation of an IDS that is both effective and specifically optimized for the IoMT context. The purpose of our work is to contribute to the body of knowledge in IoMT security, offering a novel approach that addresses the unique challenges posed by this emerging field.

The rest of the paper is organized as follows. In Section 2, related works are explored. Section 3 describes the methodology and proposed techniques. Section 4 presents and discusses the experimental results in comparison with related models. The paper ends with a concluding section that revisits the work performed and provides suggestions for future research.

## 2. Related Works

### 2.1. Deep Learning Approaches in the IoMT

The recent advancements in technology and cybersecurity are highlighted through innovative solutions proposed in five research papers, each addressing unique challenges in their respective fields. The paper [32] introduces an improved evolutionary algorithm, TA-MaEA, for optimizing hybrid microgrid systems, focusing on balancing cost, reliability, emissions, and power supply. This approach significantly reduces system costs compared to existing algorithms. In the realm of the IoT, study [33] enhances data service quality by proposing a novel heterogeneous temporal anomaly reconstruction GAN (HTA-GAN), which excels in anomaly detection and robustness using a BiGAN structure. Addressing security in networked control systems, [34] presents a dynamic event-triggered protocol and an online predictive control algorithm to maintain stability and performance under cyber-attacks and packet dropouts. The study [35] proposed a situation-aware service coordination platform based on event-driven SOA, improving efficiency and reliability in distributed IoT environments. Lastly, [36] tackles Smart Grid cybersecurity by introducing a hybrid deep learning approach for detecting DDoS attacks, achieving a high accuracy and enhancing grid reliability. Together, these studies demonstrate significant strides toward optimizing and securing critical systems like microgrids, IoT infrastructures, and Smart Grids.

The solution proposed in [24] introduces a novel deep learning-based approach to handle network-based intrusion detection in the Internet of Medical Things (IoMT) environment, aiming to address the issue of imbalanced data in the WUSTL EHMS 2020 dataset. Unlike previous works, this approach forgoes data pre-processing and augmentation, opting instead for cost-sensitive learning. This strategy attributes more weight to classes with fewer IoMT network traffic data samples and less weight to classes with a higher number of samples during model training. The proposed model is assessed on various features such as network features, patient biometrics, and a combination of both. It incorporates both Convolutional Neural Network (CNNs) and Long Short-Term Memory (LSTM) layers to robustly extract the spatial and time series features of network flow and patient biometrics. Additionally, a global attention mechanism is integrated to aid in extracting crucial features from the CNNs and LSTM layers. Finally, this research offers a comparative evaluation of the proposed model against existing intrusion detection studies within the IoMT environment, highlighting its unique contributions. 

The study [28] addresses the shortcomings of recurrent neural networks (RNNs), including their difficulties in identifying complex features of minority classes, issues with fading gradients, and the limited scalability of their LSTM and GRU variants due to their sequential computation. The authors propose enhancements to the simple recurrent unit (SRU) architecture, such as the use of bidirectional SRUs and skip connections to mitigate these problems and improve accuracy. This threat detection model’s performance is then compared to GRU and LSTM RNN variants using the ToN–IoT dataset. Furthermore, the study proposes an explainable security model for threat detection in Internet of Medical Things (IoMT) networks. This model, which the authors claim is the first of its kind, leverages explainable AI (XAI) to enable human experts to interpret the underlying reasoning and data evidence. The model’s key contributions include feature importance analysis for improved threat detection and intrusion discovery, a novel bidirectional SRU-driven deep learning model using skip connections for IoMT network security, and empirical validation of the model’s efficiency and high accuracy in identifying various cyber-threats compared to current leading methods. 

The development of an intrusion detection system (IDS) model for the Internet of Medical Things (IoMT) environment was tackled by [25]. Despite the existence of numerous data mining techniques, there are still challenges in detecting online transactions and intrusions in large data volumes. The study uses a deep neural network (DNN) to develop an IDS that can effectively classify and predict unforeseen cyber-attacks. The IDS model involves four main steps: one-hot encoding of categorical data, normalization of data using the standard scalar method, data optimization using the grey wolf optimization algorithm, and application of a DNN to the pre-processed data for classification. The model’s efficacy is compared to other state-of-the-art algorithms. This work’s contribution includes the use of the grey wolf optimization technique to improve the IDS model’s performance, a faster convergence rate in finding the global minima, a reduction in machine learning model training time, and a secured data transfer in the IoMT architecture.

### 2.2. Anomaly Detection and Architecture-Based IDSs

An anomaly-based IDS for the IoMT [5] was developed to enhance security in the Internet of Medical Things (IoMT) settings. The proposed model leverages a fog–cloud architecture and combines deep learning (DL) and machine learning (ML), creating ensemble learning, to identify abnormal communication patterns and prevent cyber-attacks. This model employs a series of Long Short-Term Memory (LSTM) networks as the first-level learner, and their outputs are used as inputs for a decision tree (DT) to distinguish between normal and attack events. Furthermore, the paper proposes a framework for deploying the IDS as Infrastructure as a Service (IaaS) in the cloud and Software as a Service (SaaS) in the fog. This system is evaluated using the ToN–IoT dataset. Key contributions of this work include a real-time IoMT traffic analysis solution and the proposed deployment of security protocols as IaaS and SaaS, addressing challenges inherent in the IoMT environment.

A multicriteria decision-making (MCDM) framework [20] for standardizing and benchmarking ML-based IDSs used in federated learning (FL) architectures of IoMT applications was proposed. This process begins by standardizing the evaluation criteria of ML-based IDSs using the fuzzy Delphi method (FDM). Subsequently, an evaluation decision matrix is formulated, based on the intersection of standardized evaluation criteria and a list of ML-based IDSs, and MCDM methods are integrated to determine the importance weights of the main and sub-standardized security and performance criteria, followed by benchmarking, and selecting the optimal ML-based IDSs. The Borda voting method is applied to combine different ranks and perform group benchmarking.

### 2.3. Early Stopping Techniques in Deep Learning Models

Early stopping is a crucial technique in deep learning to prevent overfitting and improve model generalization. It involves monitoring the model’s performance on a separate validation dataset and stopping the training process when the performance stops improving. Several studies have highlighted the significance of early stopping in deep learning models. El-Shafie and Noureldin [37] discuss the timing of early stopping, emphasizing the need for a principled approach to determine the optimal stopping point. Dong and Zhang [38] demonstrate the feasibility of obtaining accurate progress estimates more quickly through judiciously inserting extra validation points between the original ones when early stopping is allowed. Furthermore, Ada and Ugur [39] apply early stopping, a method previously used in supervised learning, to deep reinforcement learning, showcasing its versatility across different learning paradigms. Moreover, the importance of early stopping in addressing overfitting is emphasized in the literature. Forouzesh and Salehi [40] mention early stopping as one of the regularization techniques applied to avoid overfitting in deep learning architectures. Additionally, Choi and Lee [41] propose a learning strategy that involves training all samples with good initialization parameters and stopping the model using early stopping techniques to prevent overfitting. Tian and Ji [42] also mention the use of early stopping and dropout regularization to combat overfitting in deep learning models. Furthermore, the relevance of early stopping in various domains is evident. Additionally, Robissout and Zaid [43] introduced an online evaluation metric for side-channel analysis and used it to perform early stopping on existing Convolutional Neural Networks. These examples demonstrate the wide applicability of early stopping beyond traditional deep learning tasks.

### 2.4. Early Stopping Techniques in the IoT 

Effective and efficient training of deep learning-based Internet of Things (IoT) systems depends on early stopping techniques. These techniques try to lessen the computational load involved in training deep learning models for Internet of Things applications and avoid overfitting. Reviewing early stopping techniques for training deep learning-based IoT systems is highly relevant to the work of mitigating the computing burden and reducing the overhead of online training, as suggested by [44]. Furthermore, [45] reviewed deep learning-based intrusion detection systems, which is relevant because early stopping techniques are essential to the training of intrusion detection systems for Internet of Things security.

The combination of deep learning’s observational powers and reinforcement learning’s decision-making capabilities for effective cyber-attack detection in the industrial IoT was also covered by [46], underscoring the importance of efficient training techniques for IoT security applications. In their survey of machine learning and deep learning techniques for IoT security, [47] emphasized the importance of training method optimization for IoT security as well as the broad use of learning algorithms in practical applications. The effectiveness of deep learning for botnet attack detection was shown by [48], highlighting the importance of early stopping techniques in deep learning model training for IoT network security. Popoola [49] emphasized the need for efficient training strategies in IoT environments and the potential of deep learning for precisely extracting information from unprocessed sensor data in complex contexts. All things considered, early stopping techniques are crucial for training deep learning-based Internet of Things systems because they support effective model training, avoid overfitting, and lighten the computational load, all of which improve the functionality and scalability of IoT applications.

While some current DL models implement early stopping techniques, they depend on predetermined ‘patience’ parameter values, based on the epoch count and batch size [50,51,52,53]. This rigid, static approach prevents the model from adapting effectively to the diverse traits of IoMT data. A fixed ‘patience’ value may not be ideal—if it is too high, the model may risk overfitting by continuing training past the point of optimal generalization and learning noise from the training data. Conversely, a ‘patience’ value set too low could halt training prematurely, resulting in an underfit model that fails to recognize the inherent patterns in the data. Therefore, finding a balance is key, and it could be beneficial to consider dynamic strategies when determining the ‘patience’ parameter for early stopping.

## 3. The Methodology

### 3.1. A Fuzzy-Based Patience Parameter Estimation for Early Stopping of LSTM Model Training

In contrast to traditional sequential models, our model dynamically adapts the number of training epochs and batch size per epoch during training, based on how much each batch contributes to the model’s accuracy. A key innovation is our use of fuzzy logic to optimize the patience parameter, which controls when the early stopping mechanism is activated during the model’s training. 

In our approach, the early stopping mechanism commences training with arbitrary parameters and suspends the process when there are no significant improvements at both levels. This mechanism monitors one or more performance indicators during the training phase of the model, which can prompt an early termination of the training process. In our study, we monitor the loss on the validation set, and training is discontinued when no further reductions in the validation loss are detected. 

To avoid halting the training process prematurely, we have incorporated a dynamic system for establishing a patience threshold. Instead of using a static value or a simple running average of the loss differences, we have developed a fuzzy logic technique that determines the optimal patience level based on multiple inputs: the model’s accuracy, validation loss, and rate of improvement.

This fuzzy logic technique takes these inputs and, through a series of fuzzy rules and defuzzification, outputs an optimal patience level. This patience level is then used to decide when to stop the training process, providing a more dynamic and adaptive approach to early stopping. The system updates the patience level after each epoch, thereby providing a nuanced, data-driven way to determine when to cease training. This novel method therefore avoids arbitrary termination and helps to prevent both overfitting and underfitting of the model.

Let F represent the fuzzy-based technique, which receives three inputs: accuracy (A), validation loss (L), and the rate of improvement (R). These inputs are fuzzified, and their corresponding membership value (μ) is determined via the following membership functions.

(1) $$A\in \left[0,1\right]\to {\{(A}_{low};\mu_{A_{low}}),(A_{medium}{;\mu }_{A_{medium}}),(A_{high};\mu_{A_{high}})\}$$

(2) $$L\in \left[0,\infty \right]\to {\{(L}_{low};\mu_{A_{low}}),(L_{medium}{;\mu }_{A_{medium}}),(L_{high};\mu_{A_{high}})\}$$

(3) $$R\in R\to {\{(R}_{low};\mu_{R_{low}}),(R_{medium}{;\mu }_{R_{medium}}),(R_{high};\mu_{R_{high}})\}$$

The fuzzy-based patience estimation is denoted as P, which is a value de-fuzzified based on two sets with corresponding membership functions:

(4) $$\{(P_{low},\mu_{P_{low}});(P_{high},{\mu_{P}}_{high})\}c$$

This technique is controlled by two rules, R1 and R2, as follows.

(5) $$R1:\;IF\;(A\;is\;A_{good})\;and\;(L\;is\;L_{poor})\;and\;(R\;is\;R_{good})\;THEN\;(P\;is\;P_{low}).$$

(6) $$R2:\;IF\;(A\;is\;A_{poor})\;and\;(L\;is\;L_{good})\;and\;(R\;is\;R_{poor})\;THEN\;(P\;is\;P_{high}).$$

The outputs from these rules $O_{R_{i}}$ is calculated using a min–max reference method, as follows.

(7) $$O_{R_{i}}=\mu_{P_{low}}(min(\mu_{A_{good}}(A),\mu_{L_{poor}}(L),\mu_{R_{good}}(R)))\;\mathrm{for}\;\mathrm{Rule}\;1.$$

(8) $$O_{R_{i}}=\mu_{P_{high}}(min(\mu_{A_{poor}}(A),\mu_{L_{good}}(L),\mu_{R_{poor}}(R)))\;\mathrm{for}\;\mathrm{Rule}\;2.$$

So, the optimal patience value can be calculated according to the following equation:

(9) $$P=\int \left(x*\mu_{O}\left(x\right)\right)dx/\int \mu_{O}(x)dx$$

where O is the aggregated output membership function, which is obtained by taking the maximum of $O_{R_{1}}$ and $O_{R_{2}}$ at each x.

An LSTM network is composed of an input layer, several hidden layers, and an output layer. A key feature of this network is the LSTM memory cells embedded into the hidden layers. Each of these LSTM memory cells possesses three distinct gates, which collectively manage its cell state: the forget gate, the input gate, and the output gate. These gates have unique roles: (1) the forget gate determines what information should be discarded, (2) the input gate decides what information is to be incorporated, and (3) the output gate establishes what information should be emitted from the cell state. 

**Figure 1:**
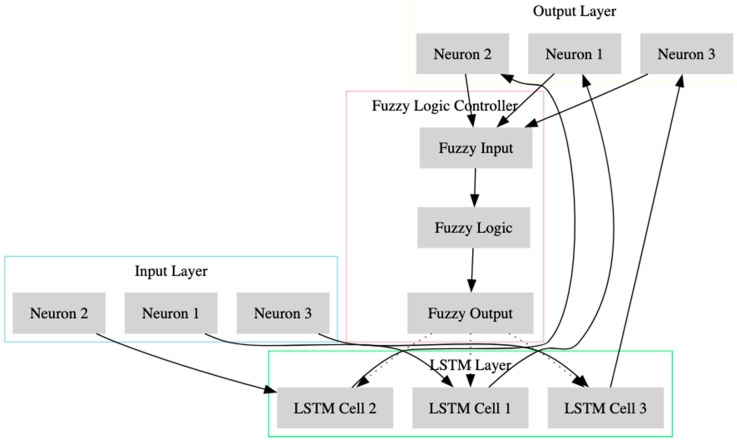
The architecture of a memory cell in an LSTM network.

The overall architecture of a memory cell is depicted in Figure 1. This diagram presents a structured view of a Long Short-Term Memory (LSTM) network integrated with a fuzzy logic controller for early stopping. The network is organized into distinct layers, each represented by color-coded blocks. The input layer, highlighted in light blue, consists of neurons that receive the initial data. This data flows into the LSTM layer, depicted in light green, where LSTM cells process the temporal aspects of the input layer. The processed information then moves to the output layer, shown in light yellow, consisting of neurons that generate the preliminary output of the network. Crucially, this output is fed into the fuzzy logic controller, colored in light pink, which comprises three parts: fuzzy input, fuzzy logic, and fuzzy output. The fuzzy logic controller evaluates the output data and applies fuzzy logic rules to determine whether the training should continue or stop early, thereby preventing overfitting and enhancing the model’s efficiency. This decision is fed back to the LSTM layer, as indicated by the dotted lines, influencing subsequent processing cycles. This integration of fuzzy logic into the LSTM network aims to optimize the training process, ensuring timely convergence and maintaining high detection accuracy, particularly in dynamic environments like the Internet of Medical Things (IoMT).

During the initial step, the forget gate’s activation values decide what information from the prior cell state needs to be discarded. Equation (9) shows such calculation:

(10) $$f_{t}=sigmoid(W_{f_{x}}x_{t}+{W_{f}}_{h}h_{t-1}+b_{f})$$

where $W_{f_{x}}$ and $W_{f_{h}}$ are weight matrices, and $f_{t}$ is the result of adding the current input $x_{t}$ at time t, the output $h_{t-1}$ from the hidden cell state at the previous time step t − 1, and the bias vector $b_{f}$. The bias vector provides the model with a greater adaptability in terms of fitting the data. The sigmoid function is used to scale the value within a range between 0 and 1, where 0 and 1 suggest that the results are interpreted as completely forgotten and completely remembered, respectively.

The next phase involves deciding the extent of updating the current time series information in the new cell state. This involves a two-step process. Firstly, candidate values ($\tilde{S}$) that may be incorporated into the new cell state ($S_{t}$) are computed using the hyperbolic tangent (tanh) function. Then, the activation values ($i_{t}$) of the input gate are calculated. These values dictate which candidate values ($\tilde{S}$) are to be included in the cell state ($S_{t}$). The calculation is as follows:

(11) $$\begin{array}{c} S=\tanh(W_{{\tilde{s}}_{x}}x_{t}+W_{{\tilde{s}}_{h}}h_{t-1}\\ +b_{{\tilde{s}}_{}}) \end{array}$$

(12) $$i_{t}=sigmoid(W_{i_{x}}x_{t}+W_{i_{h}}h_{t-1}+b_{i})$$

Then, new cell states ($S_{t}$) are generated using a combination of the prior cell state ($S_{t-1}$) and the current candidate values ($\tilde{S}$). The calculation for this is as follows:

(13) $$S_{t}=f_{t}\times S_{t-1}+i_{t}\times \tilde{S_{t}}$$

Here, the product of the previous cell state ($S_{t-1}$) and $f_{t}$ establish the amount of past information that needs to be discarded, whereas the product of the candidate values ($\tilde{S_{t}}$) and $i_{t}$ defines the volume of current information that needs to be retained. By adding the preceding results, the new cell state ($S_{t}$) is obtained.

The output ($h_{t}$) is regulated by the activation values ($O_{t}$). The calculation for this is as follows:

(14) $$O_{t}=sigmoid\;(W_{O_{x}}x_{t}+W_{o_{h}}h_{t-1}+b_{O})$$

(15) $$h_{t}=O_{t}\times tanh(S_{t})$$

The LSTM network needs sequences of input features for its training process. The network processes the sequential input at each instance (t), as expressed in the equations. Throughout the training, the weights (W) and bias terms (b) are optimized with the goal of minimizing the loss of the specified objective function.

### 3.2. The Improved Fuzzy-Based Self-Tuning LSTM Model for the IDS in the IoMT 

**Figure 2:**
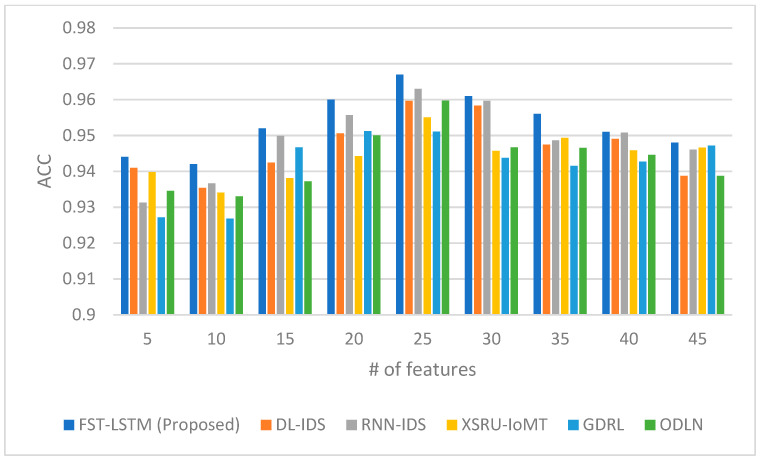
Accuracy comparison between the proposed FST-LSTM model and existing models.

The proposed fuzzy-based self-tuning LSTM model for the IDS in the IoMT (FST-LSTM), as illustrated in Figure 2, consists of two main phases: data pre-processing and model training. During data pre-processing, network flow and patient biometric data from medical sensors are transformed into numerical forms suitable for modeling. Several steps were taken during the pre-processing phase to maintain data integrity. These included normalizing the data to retain its original range, refraining from reordering the dataset to keep the time sequence intact, and avoiding any resampling operations to maintain consistent data collection intervals. Such cautious pre-processing preserves critical data characteristics, ensuring accurate subsequent analysis and results. Additionally, noise, which can come from measurement errors, missing values, or outliers, can lead to poor model performance and unreliable outputs. To tackle this issue, a filter based on the statistical mean and standard deviation was employed to identify and eliminate outliers in each attribute of the dataset.

Then, normalization was conducted to scale all attribute values between 0 and 1, which mitigates the risk of machine learning algorithms favoring attributes with larger ranges during their training. Normalization also helps minimize the effect of large values, improves the algorithm convergence, reduces overfitting, and prevents model bias toward certain features. Hence, it facilitates a more accurate depiction of the relationships among the features in the dataset. After that, we used the features selection technique proposed in [54] to select a compact set of relevant, non-redundant features, which reduces the model’s complexity. 

The next step was developing the LSTM-based model with an improved early stopping mechanism that prevents the model from overfitting and underfitting. This stage incorporates a deep learning model designed to detect attacks in IoMT network traffic. The features are fed into Long Short-Term Memory (LSTM) layers, which collaboratively learn their spatial and temporal patterns. Rather than using only the final hidden states, the LSTM model takes all hidden states into consideration and feeds them into a global attention layer similar to soft and additive attention, as described in [24]. This layer employs a Relu activation function on all of the hidden state features. These features then pass into a fully connected layer with 50 neurons. Dropout and batch normalization methods are used in the hidden layers to accelerate training. The model ultimately classifies data inputs as either normal or attack. Due to the significant imbalance in IoMT network traffic, this work adopts a cost-sensitive learning approach, assigning greater weights to the attack class and lesser weights to the normal class during model training. Initial values for the cost matrix are randomly selected following a Gaussian distribution and are fine-tuned during the training phase. 

### 3.3. Description of the Dataset

In this study, we utilized the WUSTL-EHMS-2020 dataset, which combines network flow parameters and patient biometric data. This dataset originated from an Enhanced Healthcare Monitoring System (EHMS) testbed that operates in real-time. The testbed consists of four main elements: medical monitoring sensors, a data-transmitting gateway, a network infrastructure, and a control unit with visualization capabilities. The data are collected from sensors attached to patients, transmitted through the gateway, and then sent to a dedicated server for visualization using routing and switching mechanisms. The EHMS testbed was specifically designed to gather network flow metrics and biometric data from patients. Its system includes six crucial components: a multi-sensor board, a gateway or central control hub, a data server, an intrusion detection system (IDS), a simulated attacker, and a dedicated network.

The PM4100 Six Pe Multi-Sensor Board, manufactured by Medical Expo, is equipped with four sensors that monitor important patient vitals such as electrocardiograms (ECGs), blood oxygen saturation (SpO2), body temperature, and blood pressure. The collected data is transmitted via a USB interface to a laptop running Windows, which serves as the gateway. The gateway presents the data visually through a graphical user interface (GUI), while also transmitting it to a server for further processing. The server, operating on an Ubuntu system, collects and analyzes the data, and assists in making informed medical decisions. The network infrastructure includes an Ethernet switch that connects the server, IDS, and a computer simulating attacks, with a router responsible for assigning dynamic IP addresses. The IDS relies on Argus network flow monitoring software to gather network flow metrics and biometric data, enabling important decisions about traffic packets. The simulated attacker, using Kali Linux, creates potential threats such as data spoofing or altering patient data during transmission to simulate hazards that may exist in healthcare monitoring systems.

Prior to utilization, the dataset underwent several pre-processing steps to ensure data quality and relevance. These steps included data cleaning to remove any inconsistencies or outliers, normalization to standardize the range of continuous initial variables, and feature selection to identify the most relevant attributes for intrusion detection. This pre-processing was critical in refining the dataset for optimal model training and performance.

### 3.4. Experimental Environment

The construction and performance assessment of the proposed model was executed using various software and tools, such as Python, Skfeature, TensorFlow, Keras, Scikit Learn, and NumPy. In addition, the organization of data samples, application of algorithms, and results interpretation were performed on a device equipped with an Intel(R) Core (TM) i7-4790 CPU @ 3.60 GHZ and 16 GB RAM.

In assessing the effectiveness of our model, we selected a set of performance metrics that are widely recognized in the field of intrusion detection. Accuracy (ACC) was chosen as the primary indicator of overall model performance, providing a straightforward measure of the model’s ability to correctly classify instances. However, to gain a more nuanced understanding of the model’s predictive power, we also included the false positive rate (FPR), detection rate (DR), and F1 score (F1). These metrics were selected because they offer a balanced view of the model’s performance, accounting for the costs of misclassification. The FPR is particularly important in the IoMT context, where false alarms can be costly and disruptive. The DR (also known as recall) is critical for ensuring that actual intrusions are reliably detected, and the F1 score provides a harmonic mean of precision and recall, which is useful when seeking a balance between the model’s sensitivity and specificity.

(16) $$ACC=\frac{TP+TN}{TP+TN+FP+FN}$$

(17) $$FPR=\frac{FP}{TN+FP}$$

(18) $$DR=\frac{TP}{TP+FN}$$

(19) $$F1=\frac{TP}{TP+0.5*(FP+FN)}$$

where TP, TN, FP, and FN denote true positive, true negative, false positive, and false negative, respectively.

## 4. Results and Discussion

This section discusses the outcomes of the proposed fuzzy-based self-tuning IDS (FST-LSTM) model and provides comparisons with related studies. Experimental evaluations were conducted using various Python-based packages, including SkLearn, Pandas, NumPy, and SkFeature. To evaluate the performance of our technique, multiple performance metrics were used, namely accuracy (ACC), false positive rate (FPR), detection rate (DR), and F1 score (F1). The training process for intrusion detection in the IoMT involved several steps. Initially, data pre-processing was performed, including normalization, handling missing values, and transforming the data for model training. Then, a set of relevant and non-redundant features were selected and projected onto the dataset. The dataset was then divided into training and validation sets. The training set was utilized to train the LSTM, while the validation set was used to assess its performance. The model’s parameters, such as the number of layers, neurons, activation function, and optimizer, were defined. The model architecture was subsequently trained using the training set, and adjustments were made based on prediction errors calculated via the loss function. After training, the model’s performance was evaluated using the validation set, involving metrics such as accuracy, precision, recall, and other relevant measures.

Table 1 shows the performance metrics for different numbers of features used in the training of the proposed LSTM-based IDS model.

As the number of features increases from 5 to 45, the accuracy (ACC) remains consistently high, ranging from 0.944 to 0.967. The false positive rate (FPR) decreases gradually, indicating a reduction in the number of false alarms, with the lowest value observed at 0.104 for 25 features. The detection rate (DR) also shows a gradual improvement, reaching a peak of 0.943 for 25 features. The F1 score, which considers both precision and recall, increases as the number of features increases, with the highest value of 0.966 achieved for 25 features. Overall, the results demonstrate that increasing the number of features has a positive impact on the performance of the LRGU-MIFS technique, leading to higher accuracy and improved detection rates while maintaining a low false positive rate.

The results in Table 1 provide evidence of the model’s sustained high performance, demonstrating the effectiveness of the fuzzy logic in determining the optimal patience value for an improved self-tuning capability. This can be attributed to the integration of fuzzy logic within the self-tuning mechanism, which accurately estimates the value of the patience parameter during the training phase. By dynamically adjusting the number of epochs and preventing overfitting, our model successfully avoids both underfitting due to insufficient epochs and overfitting caused by excessive training. This robust approach contributes to the reliable detection rate of our fuzzy-based self-tuning LSTM IDS, highlighting its ability to effectively identify a significant proportion of actual intrusions within the IoMT environment. Through the effective capture and classification of anomalous patterns, the proposed IDS has the ability to ensure the security and integrity of healthcare systems, protecting them from potential threats.

The results presented in Table 1 also demonstrate interesting performance trends, particularly when the number of features reaches 25. At this point, there is a noticeable improvement in the model’s performance across multiple evaluation metrics. The accuracy (ACC) increases to 0.967, indicating a high level of correct classifications. Additionally, the false positive rate (FPR) significantly decreases to 0.104, indicating a reduced number of false alarms. The detection rate (DR) remains consistently high at 0.943, indicating the model’s ability to accurately identify intrusions. The F1 score (F1) also reaches a high value of 0.966, reflecting the model’s balanced precision and recall. These performance enhancements at 25 features suggest that the proposed model was able to perceive the attack patterns even with a fewer number of epochs and fewer features used as inputs. Such a level of performance suggests that the early stopping mechanism maintained a good trade-off between performance and complexity, allowing the model to achieve high accuracy without sacrificing efficiency.

**Figure 3:**
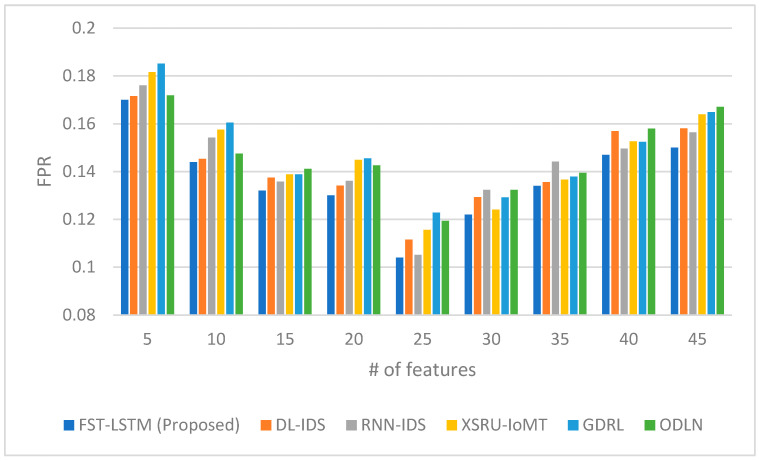
False positive rate comparison between the proposed FST-LSTM model and existing models.

**Figure 4:**
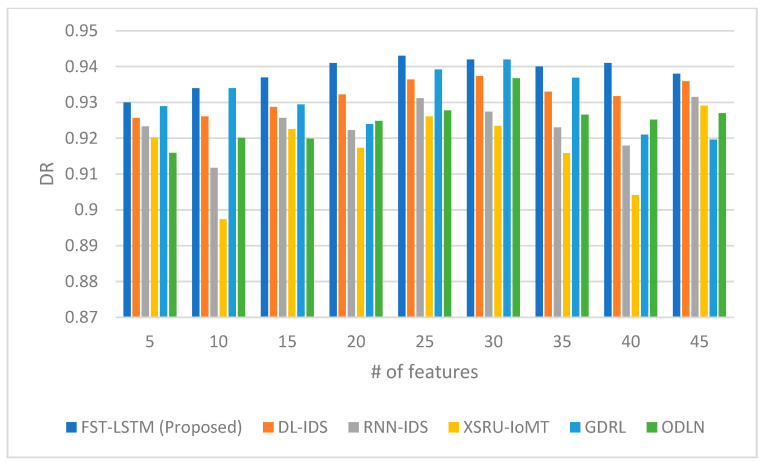
Detection rate comparison between the proposed FST-LSTM model and existing models.

**Figure 5:**
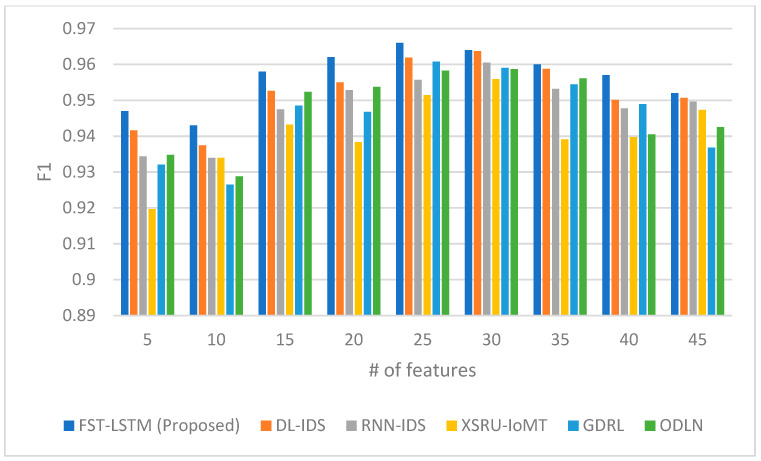
F1 score comparison between the proposed FST-LSTM model and existing models.

The performance of the proposed FST-LSTM model is compared with three existing models, namely the DL-IDS [24], RNN-IDS [55], XSRU-IoMT [25], GDRL [5], and ODLN [38], across multiple evaluation metrics, as shown in Figure 2, Figure 3, Figure 4 and Figure 5. The rationale for choosing these studies to compare ours with is that they work on IoMT data and apply deep learning algorithms for developing the IDS. Figure 2 presents the accuracy scores, indicating the proportion of correct classifications. The FST-LSTM model consistently outperforms the other LSTM models across different numbers of features, achieving the highest accuracy scores. The proposed model shows a high accuracy, peaking at 25 features with a score of 0.967. Beyond 30 features, a slight decline in accuracy is observed for most models, including the FST-LSTM, suggesting a potential threshold for optimal feature utilization.

The comparison results in the table reveal the FST-LSTM model as a robust performer in the IoMT IDS landscape, consistently outperforming other models, especially in scenarios with a higher number of features. Its peak performance at 25 features suggests an optimal balance between feature count and model efficiency. The decline in accuracy beyond this point for the FST-LSTM and other models implies a potential overfitting or diminishing of returns with too many features. Comparatively, models like the XSRU-IoMT show close competition, especially at higher feature counts, while the GDRL and ODLN lag slightly behind across most feature ranges. These results underscore the importance of feature selection in IDS model performance, highlighting that an increased number of features does not always correlate with enhanced detection capabilities, particularly beyond a certain threshold.

Similar trends can be observed in Figure 5, which presents the F1 scores, reflecting the balance between precision and recall. The FST-LSTM model consistently achieves the highest F1 scores, indicating its superior performance in capturing both true positives and true negatives. The proposed model demonstrates a consistent increase in F1 score as the number of features grows from 5 to 25, peaking at 0.966 for 25 features. Beyond this point, a gradual decrease in F1 score is observed for the FST-LSTM and other models, indicating a potential limit to the effectiveness of increasing feature counts. Comparison reveals that the FST-LSTM model is a highly effective solution concerning the IoMT IDS, maintaining superior performance across a wide range of feature counts. Its peak F1 score at 25 features suggests an optimal point for feature utilization, balancing precision and recall effectively. The gradual decline in F1 scores beyond 25 features for all models, including the FST-LSTM, suggests a potential overfitting issue or inefficiency in handling an excessive number of features. In comparison, models like the DL-IDS and RNN-IDS show competitive performances, especially at higher feature counts, closely following the FST-LSTM model. The XSRU-IoMT, GDRL, and ODLN models exhibit varying degrees of effectiveness, with some performing better at lower feature counts and others at higher. These results highlight the importance of an appropriate feature count in maximizing the F1 measure, indicating that an excessive number of features might lead to a decrease in the balance between precision and recall.

Figure 3 shows the false positive rates (FPRs), wherein the FST-LSTM model consistently exhibits lower FPR values compared to the other models, indicating its ability to reduce false alarms. The proposed model demonstrates an overall decreasing trend in false positive rates as the number of features increases, with a notable dip at 25 features (0.104). Beyond 25 features, there is a gradual increase in false positive rates for the FST-LSTM and other models, suggesting a limit to the effectiveness of feature count in reducing false alarms. Analysis of the false positive rate indicates that the FST-LSTM model is effective in minimizing incorrect threat detections, especially in scenarios with a moderate number of features. Its lowest false positive rate at 25 features suggests an optimal balance in feature count, where the model efficiently distinguishes between normal and malicious activities. The increase in false positive rates beyond this point for the FST-LSTM and other models implies a potential overfitting or reduced efficiency with too many features. Compared to other models, the FST-LSTM generally maintains a lower false positive rate, indicating its superior capability to avoid false alarms. Other models, such as the DL-IDS and RNN-IDS, show competitive performances but slightly higher false positive rates at various feature counts. The XSRU-IoMT, GDRL, and ODLN models exhibit higher false positive rates, particularly at higher feature counts, indicating a potential difficulty to maintain accuracy without compromising on false detections. These results highlight the importance of a balanced feature selection in IDS models for the IoMT, where an excessive number of features might lead to increased false alarms, undermining the system’s reliability and user trust.

Lastly, Figure 4 presents the detection rates, measuring the ability to accurately identify intrusions. Again, the FST-LSTM model demonstrates higher detection rates across different numbers of features, showcasing its effectiveness in identifying intrusions in the IoMT environment. The proposed model exhibits a steady increase in detection rate as the number of features increases from 5 to 25, reaching a peak at 0.943 for 25 features. Beyond 30 features, the detection rate for the FST-LSTM and other models shows a slight fluctuation, suggesting a plateau in performance improvement with an increase in feature count. Comparison reveals that the FST-LSTM model is a consistently strong performer in detecting intrusions within the IoMT environment. Its peak performance at 25 features indicates an optimal balance in utilizing a sufficient number of features to effectively identify threats. The slight fluctuations in detection rate beyond 30 features across all models, including the FST-LSTM, hint at a potential limit to the benefits of increasing the feature counts, where additional features may not significantly enhance detection capabilities. Compared to other models, the FST-LSTM generally maintains a higher detection rate, especially in the mid-range of feature counts. Models like the DL-IDS and RNN-IDS show competitive performances, closely following the FST-LSTM model, while the XSRU-IoMT, GDRL, and ODLN models exhibit varying effectiveness at different feature counts. These results underscore the importance of an optimal feature selection strategy in IDS models for the IoMT, where too many features might not necessarily lead to improved detection rates and could potentially introduce complexity without significant benefits.

These results highlight the superior performance of the proposed FST-LSTM model compared to the existing LSTM models, suggesting its efficacy in intrusion detection tasks.

To mitigate overfitting and underfitting in our LSTM model and better its performance, we have implemented a multifaceted approach that leverages both algorithmic and architectural strategies. Algorithmically, our model employs a fuzzy-based dynamic adjustment of the patience parameter in the early stopping mechanism. This approach is preferred over static early stopping because it allows the model to adaptively determine the optimal point at which to halt training based on the actual learning progress, rather than a predetermined, fixed number of epochs. The fuzzy logic system evaluates the model’s performance, considering accuracy, validation loss, and the rate of improvement, to dynamically adjust the patience parameter. This ensures that the model continues to learn as long as significant improvements are made, thereby avoiding premature stopping (which could lead to underfitting) and excessive training (which could lead to overfitting).

Architecturally, we introduced dropout layers and batch normalization within the hidden layers of our LSTM network. Dropout layers randomly deactivate a subset of neurons during the training process, which prevents the network from becoming overly dependent on any specific neuron and thus reduces overfitting. Batch normalization standardizes the inputs to a layer for each mini-batch, stabilizing the learning process and accelerating convergence by reducing internal covariate shift. These combined strategies form a robust defense against overfitting and underfitting, ensuring that our model achieves a balance between bias and variance, ultimately leading to better generalization of unseen data. Our choice of these specific techniques is driven by their proven effectiveness in similar contexts, as documented in the literature, and their suitability for the complex and dynamic nature of IoMT network traffic and attack patterns.

## 5. Future Research Directions

The self-tuning mechanism of our fuzzy-based LSTM model presents a novel approach to intrusion detection in the Internet of Medical Things (IoMT), yet there is ample room for further refinement and exploration. Future research could delve into optimizing the fuzzy logic rules and membership functions to enhance the model’s responsiveness and accuracy. Additionally, investigating adaptive self-tuning techniques that can adjust not only the patience parameter but also other hyperparameters such as learning rate and dropout rate in real-time could lead to more sophisticated and finely tuned models. Another promising direction is the application of self-tuning mechanisms to different types of neural network architectures that are gaining traction in the IoMT, such as attention-based models or transformer networks. These architectures could potentially benefit from the dynamic adjustment capabilities of self-tuning models, particularly in handling the vast and varied data streams inherent in IoMT environments.

Moreover, the integration of self-tuning models with online learning paradigms could be explored to facilitate continuous learning and adaptation to new and evolving attack patterns without the need for retraining the model from scratch. This could significantly enhance the model’s longevity and effectiveness in real-world applications. The potential for self-tuning mechanisms to reduce computational overhead and energy consumption in IoMT devices also presents an important research opportunity. By optimizing the training process, such mechanisms could enable a more efficient deployment of advanced IDSs on resource-constrained devices, thus broadening the reach and scalability of secure IoMT solutions.

Lastly, the development of benchmarking frameworks to evaluate the performance of self-tuning models against traditional and static approaches in various IoMT scenarios would provide valuable insights and drive innovation in the field. Such frameworks could help in systematically assessing the impact of self-tuning on the overall efficacy and efficiency of intrusion detection systems. By focusing on these areas, future research can significantly advance the state of the art in self-tuning intrusion detection systems, paving the way for more autonomous, reliable, and efficient security solutions in the IoMT landscape.

## 6. Conclusions

In conclusion, our research introduced a novel fuzzy-based self-tuning LSTM intrusion detection system (IDS) specifically designed for the Internet of Medical Things (IoMT). The model’s dynamic adjustment of training epochs and implementation of early stopping have proven to be highly effective, as evidenced by our experimental results, which indicate superior performance over existing IDS models in key metrics such as accuracy, false positive rate, detection rate, and F1 score. Importantly, these advancements in intrusion detection directly contribute to the broader IoMT context by enhancing the security framework essential for reliable healthcare operations. The ability of our system to accurately and efficiently detect intrusions ensures that healthcare providers can maintain the integrity and availability of critical medical devices and patient data. This, in turn, is fundamental to patient safety, as secure IoMT devices are less likely to suffer from disruptions that could lead to adverse health outcomes. While our findings mark a significant step forward, we acknowledge that the landscape of IoMT security is ever-evolving, with numerous opportunities for further research. Future work could refine the application of fuzzy logic in early stopping to optimize both the performance and convergence speed of IDS models. Additionally, integrating a broader array of data sources and employing advanced machine learning techniques could further bolster the IDS’s ability to pre-empt sophisticated cyber-threats. Extending our model to handle real-time data more effectively will also be crucial in minimizing response times to potential attacks, thereby safeguarding the continuity of healthcare services. Lastly, as the IoMT continues to grow, ongoing research must address the challenge of advancing security measures to protect against new threats and ensure the privacy of sensitive health data. By continuing to develop robust and adaptive security solutions, we can better protect the IoMT infrastructure, which is so integral to modern healthcare delivery and patient safety.

## Figures and Tables

**Table 1:** The performance evaluation of the proposed model in terms of accuracy (ACC), false positive rate (FPR), detection rate (DR), and F1 score (F1).

No. of Features	ACC	FPR	DR	F1
5	0.944	0.17	0.93	0.947
10	0.942	0.144	0.934	0.943
15	0.952	0.132	0.937	0.958
20	0.960	0.13	0.941	0.962
25	0.967	0.104	0.943	0.966
30	0.961	0.122	0.942	0.964
35	0.956	0.134	0.94	0.96
40	0.951	0.147	0.941	0.957
45	0.948	0.15	0.938	0.952

## Data Availability

Data are contained within the article.

## References

[1] Rbah Y., Mahfoudi M., Balboul Y., Fattah M., Mazer S., Elbekkali M., Bernoussi B. Machine learning and deep learning methods for intrusion detection systems in iomt: A survey. Proceedings of the 2022 2nd International Conference on Innovative Research in Applied Science, Engineering and Technology (IRASET).

[2] Otoum Y., Wan Y., Nayak A. Federated transfer learning-based ids for the internet of medical things (iomt). Proceedings of the 2021 IEEE Globecom Workshops (GC Wkshps).

[3] Maseer Z.K., Yusof R., Mostafa S.A., Bahaman N., Musa O., Al-rimy B.A.S. (2021). DeepIoT. IDS: Hybrid deep learning for enhancing IoT network intrusion detection. Comput. Mater. Contin..

[4] Somasundaram R., Thirugnanam M. (2021). Review of security challenges in healthcare internet of things. Wirel. Netw..

[5] Khan F., Jan M.A., Alturki R., Alshehri M.D., Shah S.T., ur Rehman A. (2023). A Secure Ensemble Learning-Based Fog-Cloud Approach for Cyberattack Detection in IoMT. IEEE Trans. Ind. Inform..

[6] Abbas S., Sampedro G.A., Abisado M., Almadhor A., Yousaf I., Hong S.-P. (2023). Harris-Hawk-Optimization-Based Deep Recurrent Neural Network for Securing the Internet of Medical Things. Electronics.

[7] Urooj U., Maarof M.A.B., Al-rimy B.A.S. A proposed adaptive pre-encryption crypto-ransomware early detection model. Proceedings of the 2021 3rd International Cyber Resilience Conference (CRC).

[8] Alghofaili Y., Albattah A., Alrajeh N., Rassam M.A., Al-rimy B.A.S. (2021). Secure cloud infrastructure: A survey on issues, current solutions, and open challenges. Appl. Sci..

[9] Ali A., Al-Rimy B.A.S., Alsubaei F.S., Almazroi A.A., Almazroi A.A. (2023). HealthLock: Blockchain-Based Privacy Preservation Using Homomorphic Encryption in Internet of Things Healthcare Applications. Sensors.

[10] Kumar A.K., Vadivukkarasi K., Dayana R. A Novel Hybrid Deep Learning Model for Botnet Attacks Detection in a Secure IoMT Environment. Proceedings of the 2023 International Conference on Intelligent Systems for Communication, IoT and Security (ICISCoIS).

[11] Saranya T., Sridevi S., Deisy C., Chung T.D., Khan M.A. (2020). Performance analysis of machine learning algorithms in intrusion detection system: A review. Procedia Comput. Sci..

[12] Bhushan B., Kumar A., Agarwal A.K., Kumar A., Bhattacharya P., Kumar A. (2023). Towards a Secure and Sustainable Internet of Medical Things (IoMT): Requirements, Design Challenges, Security Techniques, and Future Trends. Sustainability.

[13] Khan I.A., Pi D. (2023). Explainable Learning Machines for Securing the IoMT Networks. The Internet of Medical Things (IoMT) and Telemedicine Frameworks and Applications.

[14] Kamalov F., Pourghebleh B., Gheisari M., Liu Y., Moussa S. (2023). Internet of medical things privacy and security: Challenges, solutions, and future trends from a new perspective. Sustainability.

[15] Ghubaish A., Salman T., Zolanvari M., Unal D., Al-Ali A., Jain R. (2020). Recent advances in the internet-of-medical-things (IoMT) systems security. IEEE Internet Things J..

[16] Si-Ahmed A., Al-Garadi M.A., Boustia N. (2023). Survey of Machine Learning based intrusion detection methods for Internet of Medical Things. Appl. Soft Comput..

[17] Al-rimy B.A.S., Maarof M.A., Shaid S.Z.M. (2019). Crypto-ransomware early detection model using novel incremental bagging with enhanced semi-random subspace selection. Future Gener. Comput. Syst..

[18] Aboaoja F.A., Zainal A., Ghaleb F.A., Al-rimy B.A.S. Toward an ensemble behavioral-based early evasive malware detection framework. Proceedings of the 2021 International Conference on Data Science and Its Applications (ICoDSA).

[19] Avinashiappan A., Mayilsamy B. (2021). Internet of medical things: Security threats, security challenges, and potential solutions. Internet of Medical Things: Remote Healthcare Systems and Applications.

[20] Alamleh A., Albahri O., Zaidan A., Albahri A., Alamoodi A., Zaidan B., Qahtan S., Alsatar H., Al-Samarraay M.S., Jasim A.N. (2022). Federated learning for IoMT applications: A standardisation and benchmarking framework of intrusion detection systems. IEEE J. Biomed. Health Inform..

[21] Saba T., Rehman A., Sadad T., Kolivand H., Bahaj S.A. (2022). Anomaly-based intrusion detection system for IoT networks through deep learning model. Comput. Electr. Eng..

[22] Khan A.R., Kashif M., Jhaveri R.H., Raut R., Saba T., Bahaj S.A. (2022). Deep learning for intrusion detection and security of Internet of things (IoT): Current analysis, challenges, and possible solutions. Secur. Commun. Netw..

[23] Ahmad R., Alsmadi I., Alhamdani W., Tawalbeh L.A. (2022). A comprehensive deep learning benchmark for IoT IDS. Comput. Secur..

[24] Ravi V., Pham T.D., Alazab M. (2023). Deep Learning-Based Network Intrusion Detection System for Internet of Medical Things. IEEE Internet Things Mag..

[25] RM S.P., Maddikunta P.K.R., Parimala M., Koppu S., Gadekallu T.R., Chowdhary C.L., Alazab M. (2020). An effective feature engineering for DNN using hybrid PCA-GWO for intrusion detection in IoMT architecture. Comput. Commun..

[26] Kumar M., Kumar A., Verma S., Bhattacharya P., Ghimire D., Kim S.-h., Hosen A.S. (2023). Healthcare Internet of Things (H-IoT): Current Trends, Future Prospects, Applications, Challenges, and Security Issues. Electronics.

[27] Al-Hawawreh M., Hossain M.S. (2023). A privacy-aware framework for detecting cyber attacks on internet of medical things systems using data fusion and quantum deep learning. Inf. Fusion.

[28] Khan I.A., Moustafa N., Razzak I., Tanveer M., Pi D., Pan Y., Ali B.S. (2022). XSRU-IoMT: Explainable simple recurrent units for threat detection in Internet of Medical Things networks. Future Gener. Comput. Syst..

[29] Alizadehsani R., Roshanzamir M., Izadi N.H., Gravina R., Kabir H.D., Nahavandi D., Alinejad-Rokny H., Khosravi A., Acharya U.R., Nahavandi S. (2023). Swarm intelligence in internet of medical things: A review. Sensors.

[30] Chaganti R., Azrour M., Vinayakumar R., Naga V., Dua A., Bhushan B. (2022). A Particle Swarm Optimization and Deep Learning Approach for Intrusion Detection System in Internet of Medical Things. Sustainability.

[31] Kumar P., Gupta G.P., Tripathi R. (2021). An ensemble learning and fog-cloud architecture-driven cyber-attack detection framework for IoMT networks. Comput. Commun..

[32] Cao B., Dong W., Lv Z., Gu Y., Singh S., Kumar P. (2020). Hybrid Microgrid Many-Objective Sizing Optimization With Fuzzy Decision. IEEE Trans. Fuzzy Syst..

[33] Chen P., Liu H., Xin R., Carval T., Zhao J., Xia Y., Zhao Z. (2022). Effectively Detecting Operational Anomalies In Large-Scale IoT Data Infrastructures By Using A GAN-Based Predictive Model. Comput. J..

[34] Li B., Zhou X., Ning Z., Guan X., Yiu K.-F.C. (2022). Dynamic event-triggered security control for networked control systems with cyber-attacks: A model predictive control approach. Inf. Sci..

[35] Cheng B., Zhu D., Zhao S., Chen J. (2016). Situation-Aware IoT Service Coordination Using the Event-Driven SOA Paradigm. IEEE Trans. Netw. Serv. Manag..

[36] AlHaddad U., Basuhail A., Khemakhem M., Eassa F.E., Jambi K. (2023). Ensemble Model Based on Hybrid Deep Learning for Intrusion Detection in Smart Grid Networks. Sensors.

[37] El-Shafie A., Noureldin A. (2011). Generalized Versus Non-Generalized Neural Network Model for Multi-Lead Inflow Forecasting at Aswan High Dam. Hydrol. Earth Syst. Sci..

[38] Dong Q., Zhang X., Luo G. (2022). Improving the Accuracy of Progress Indication for Constructing Deep Learning Models. Ieee Access.

[39] Ada S.E., Ugur E., Akin H.L. (2022). Generalization in transfer learning: Robust control of robot locomotion. Robotica.

[40] Forouzesh M., Salehi F., Thiran P. Generalization Comparison of Deep Neural Networks via Output Sensitivity. Proceedings of the 2020 25th International Conference on Pattern Recognition (ICPR).

[41] Choi H., Lee H. (2023). Exploiting All Samples in Low-Resource Sentence Classification: Early Stopping and Initialization Parameters. IEEE Access.

[42] Tian C., Ji W. (2017). Auxiliary Multimodal LSTM for Audio-Visual Speech Recognition and Lipreading. arXiv.

[43] Robissout D., Zaid G., Colombier B., Bossuet L., Habrard A. (2020). Online Performance Evaluation of Deep Learning Networks for Profiled Side-Channel Analysis. Constructive Side-Channel Analysis and Secure Design: 11th International Workshop, COSADE 2020, Lugano, Switzerland, 1–3 April 2020, Revised Selected Papers 11.

[44] Lee I. (2019). The Internet of Things for enterprises: An ecosystem, architecture, and IoT service business model. Internet Things.

[45] Li H., Ota K., Dong M. (2018). Learning IoT in edge: Deep learning for the Internet of Things with edge computing. IEEE Netw..

[46] Ferrag M.A., Maglaras L., Ahmim A., Derdour M., Janicke H. (2020). Rdtids: Rules and decision tree-based intrusion detection system for internet-of-things networks. Future Internet.

[47] Ashraf N., Sheikh S.A., Khan S.A. (2022). Performance analysis of SWIPT assisted cooperative Internet of Things (IoT) network under Optimal and Adaptive Power Splitting Schemes. Internet Things.

[48] Al-Garadi M.A., Mohamed A., Al-Ali A.K., Du X., Ali I., Guizani M. (2020). A survey of machine and deep learning methods for internet of things (IoT) security. IEEE Commun. Surv. Tutor..

[49] Popoola S.I. (2022). Federated Deep Learning for Botnet Attack Detection in IoT Networks. Ph.D. Thesis.

[50] Vu L., Nguyen Q.U., Nguyen D.N., Hoang D.T., Dutkiewicz E. (2020). Deep transfer learning for IoT attack detection. IEEE Access.

[51] Haq M.A., Rahim Khan M.A. (2022). DNNBoT: Deep neural network-based botnet detection and classification. Comput. Mater. Contin..

[52] Ullah I., Mahmoud Q.H. An Anomaly Detection Model for IoT Networks based on Flow and Flag Features using a Feed-Forward Neural Network. Proceedings of the 2022 IEEE 19th Annual Consumer Communications & Networking Conference (CCNC).

[53] Ge M., Syed N.F., Fu X., Baig Z., Robles-Kelly A. (2021). Towards a deep learning-driven intrusion detection approach for Internet of Things. Comput. Netw..

[54] Alalhareth M., Hong S.-C. (2023). An Improved Mutual Information Feature Selection Technique for Intrusion Detection Systems in the Internet of Medical Things. Sensors.

[55] Saheed Y.K., Arowolo M.O. (2021). Efficient Cyber Attack Detection on the Internet of Medical Things-Smart Environment Based on Deep Recurrent Neural Network and Machine Learning Algorithms. IEEE Access.

